# Occurrence of ^210^Po and Biological Effects of Low-Level Exposure: The Need for Research

**DOI:** 10.1289/ehp.1104607

**Published:** 2012-04-26

**Authors:** Ralph L. Seiler, Joseph L. Wiemels

**Affiliations:** 1 Environmental Sciences Graduate Program, University of Nevada–Reno, Reno, Nevada, USA; 2 Laboratory for Molecular Epidemiology, University of California at San Francisco Helen Diller Comprehensive Cancer Center, San Francisco, California, USA

**Keywords:** cigarettes, drinking water, Fallon, human health, lead-210, leukemia, ovary, polonium-210

## Abstract

Background: Polonium-210 (^210^Po) concentrations that exceed 1 Bq/L in drinking-water supplies have been reported from four widely separated U.S. states where exposure to it went unnoticed for decades. The radionuclide grandparents of ^210^Po are common in sediments, and segments of the public may be chronically exposed to low levels of ^210^Po in drinking water or in food products from animals raised in contaminated areas.

Objectives: We summarized information on the environmental behavior, biokinetics, and toxicology of ^210^Po and identified the need for future research.

Methods: Potential linkages between environmental exposure to ^210^Po and human health effects were identified in a literature review.

Discussion: ^210^Po accumulates in the ovaries where it kills primary oocytes at low doses. Because of its radiosensitivity and tendency to concentrate ^210^Po, the ovary may be the critical organ in determining the lowest injurious dose for ^210^Po. ^210^Po also accumulates in the yolk sac of the embryo and in the fetal and placental tissues. Low-level exposure to ^210^Po may have subtle, long-term biological effects because of its tropism towards reproductive and embryonic and fetal tissues where exposure to a single alpha particle may kill or damage critical cells. ^210^Po is present in cigarettes and maternal smoking has several effects that appear consistent with the toxicology of ^210^Po.

Conclusions: Much of the important biological and toxicological research on ^210^Po is more than four decades old. New research is needed to evaluate environmental exposure to ^210^Po and the biological effects of low-dose exposure to it so that public health officials can develop appropriate mitigation measures where necessary.

In 2007, very high levels of the naturally occurring radioisotope polonium-210 (^210^Po) were discovered in drinking water from numerous private wells in Lahontan Valley, Churchill County, in northern Nevada. Of the 60 private wells and 3 public supply wells sampled in the valley (Seiler 2011), more than one-third of the private wells had ^210^Po levels exceeding 0.55 Bq/L, and 10% had levels that exceeded 2 Bq/L. This level is extremely unusual—fewer than 100 wells in the United States have reported ^210^Po activities that exceed 0.55 Bq/L, and the maximum value measured in Lahontan Valley, 6.59 Bq/L, is the fourth highest value reported in the United States (Seiler 2012).

^210^Po is one of the most toxic substances known because of its intense radioactivity, with 1 µg of ^210^Po having an activity of 1.66 × 10^8^ Bq. After the death of Alexander Litvinenko from ^210^Po poisoning in 2006, several papers were published that addressed the diagnosis, treatment, and toxicity of acute ^210^Po poisoning (Harrison et al. 2007; Jefferson et al. 2009; Scott 2007) and the need for medical toxicology expertise about ^210^Po because of its potential use in radiation terrorism events (Nemhauser 2010). The estimated acute lethal dose from oral ingestion for an adult if untreated is 10–30 µg (Jefferson et al. 2009), and as little as 1 µg might be lethal to the most radiosensitive members of the population (Scott 2007).

As an alpha-particle–emitting decay product of radium-226 (^226^Ra), ^210^Po is classified as a Group 1 human carcinogen [International Agency for Research on Cancer (IARC) 2001]. Numerous epidemiological studies, which were reviewed by Canu et al. (2011), have investigated associations between exposure to members of the uranium-238 (^238^U) decay series in drinking water and cancers such as leukemia (e.g., Hoffmann et al. 1993; Lyman et al. 1985; Raaschou-Nielsen et al. 2008). Because ^210^Po is the last radioactive member of the ^238^U decay series, a common feature of all these studies is the presence of ^210^Po in the environment, even if it was not measured. Because ^210^Po is a human carcinogen and because information on environmental exposure to it is poorly known, we reviewed the literature on ^210^Po.

In this paper, we summarize relatively unknown and disparate information on the environmental behavior, biokinetics, and toxicology of ^210^Po and suggest supportive research that could help guide future investigations.

## Environmental Behavior of ^210^Po

**Figure 1:**
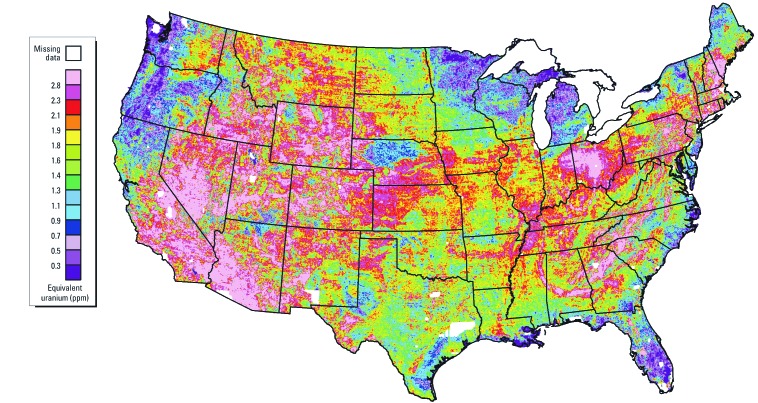
Equivalent uranium concentrations in surface soils and rocks of the conterminous United States. Adapted from Duval et al. (2005).

^210^Po has a relatively short half-life of 138.4 days and decays to lead-206 (^206^Pb) by emitting an alpha particle. It is the last unstable isotope in the ^238^U decay series and is present in the environment wherever ^238^U or any other members of the ^238^U decay series, such as ^226^Ra, radon, or ^210^Pb, are present. The average crustal U concentration is approximately 2.7 mg/kg (Siegel and Bryan 2003), and > 99% of natural U is ^238^U (Bordujenko 2002). Large areas in the United States have surficial uranium concentrations exceeding 2.7 mg/kg (Figure 1). The specific activity of ^238^U is 12.4 Bq/mg, and in old sediments where radon is not lost to the atmosphere, the activity of ^210^Po in secular equilibrium with 2.7 mg/kg of ^238^U would be about 33.5 Bq/kg.

The main source of ^210^Po in the atmosphere and oceans is from the emanation of radon gas from surficial sediments to the atmosphere where it is spread by diffusion and winds (Parfenov 1974; Persson and Holm 2011). In addition, high ^210^Po can be introduced into the atmosphere in volcanic emissions because of the high volatility of ^210^Po compared with ^210^Pb (Kim et al. 2011). Radon’s decay products, ^210^Pb and ^210^Po, have strong affinities for particles and become widely distributed on the earth’s surface as the ^210^Pb and ^210^Po that are formed from decay of radon in the atmosphere adsorb to aerosol particles and fall onto the land or sea.

^210^Po activities exceeding 1 Bq/L have been found in private wells in Florida and Nevada and public supply wells in Louisiana and Maryland (Seiler et al. 2011). ^210^Po in groundwater is normally < 30 mBq/L because it strongly adsorbs to aquifer materials (Harada et al. 1989). In typical aquifer sediments, however, ^210^Po concentrations are high enough that geochemical and microbial processes that mobilize only a small percentage of the ^210^Po that is present can yield groundwater with ^210^Po concentrations that greatly exceed safe levels in drinking water. Wells with high ^210^Po in Florida are typically shallow wells located in the central part of the state (Harada et al. 1989) where the ultimate source of the ^210^Po is uranium-rich phosphate rock in the aquifers and phosphogypsum waste products from mining operations (Cherrier et al. 1995). U concentrations in aquifer sediments near contaminated wells in Nevada range from 1.4 to 4.5 mg/kg (Seiler et al. 2011) and are typical of the western United States (Figure 1). U concentrations in sediments near contaminated wells in Maryland and Louisiana have not been described.

A comparison of naturally contaminated wells in the United States and Finland (Seiler et al. 2011) suggested that, in areas where ^238^U and radon are present in the environment, elevated ^210^Po activities in groundwater may be found in two distinct groundwater environments, one with oxic or anoxic groundwater with extremely high radon levels, and the other with anoxic, sulfate-reducing groundwater with low hydrogen sulfide concentrations and low-to-high radon levels. In wells in Florida, Maryland, and Nevada, aqueous ^210^Pb concentrations were much less than ^210^Po concentrations (Harada et al. 1989; Outola et al. 2008; Seiler 2011), indicating that the ^210^Po was being mobilized from the sediments. Aqueous ^210^Po concentrations were the same as ^210^Pb concentrations in the Finnish wells (Seiler et al. 2011)—a finding that indicates secular equilibrium between the two radioisotopes and that the ^210^Po was derived from the decay of ^210^Pb in the water. Elevated ^210^Po levels in Finnish wells were associated with radon activities up to 43,000 Bq/L (Seiler et al. 2011). A Maryland well that had a ^210^Po activity of 1.7 Bq/L did not contain high radon (only 8.5 Bq/L) (Outola et al. 2008), an indication that elevated ^210^Po does not require the presence of elevated radon.

## ^210^Po Biokinetics and Biodistribution after Ingestion

*Absorption and excretion.* The fraction of ^210^Po absorbed from the digestive tract into the blood (f1) ranges from 3% to 80% (Harrison et al. 2007). Experimental data indicate greater absorption of biologically incorporated ^210^Po than of inorganic ^210^Po (Haines et al. 1993). In some areas, caribou and shellfish are important sources of ^210^Po in the diet of humans. Thomas et al. (2001) found that the average f1 was 56% among 13 volunteers who consumed caribou meat, and Hunt and Rumney (2007) reported an average f1 of approximately 51% among five volunteers who consumed shellfish. In a review article, Harrison et al. (2007) cited three different studies that measured f1 in rats that had orally ingested different Po compounds. The results showed that the f1 was 6% for Po chloride, 5% for Po nitrate, and 7–9% for Po citrate. The f1 of ^210^Po is unknown for ingestion of naturally contaminated groundwater, where the ^210^Po may be present in volatile organic forms such as dimethyl polonium (Hussain et al. 1995), in hydrophilic complexes with fulvic acids (Oural et al. 1988), or as an anionic colloid (Seiler et al. 2011). With prolonged chronic intake, absorption of ^210^Po through the digestive tract may increase sharply (Moroz and Parfenov 1972), possibly because of damage to the intestinal wall through persistent bombardment by alpha particles.

Following exposure to ^210^Po, much more ^210^Po is excreted in feces than in urine (Moroz and Parfenov 1972). Renal excretion of ^210^Po is slow compared with other elements because it binds strongly to hemoglobin and plasma proteins and is not filtered by the kidneys (Thomas et al. 2001). ^210^Po is also excreted through the cutaneous glands and the hair (Moroz and Parfenov 1972) and in milk (Parfenov 1974). In an experiment that used a lactating goat, Schreckhise and Watters (1969) estimated the transfer coefficient (percent intake by the goat per liter of milk produced) for ^210^Po was 0.18%.

Hunt and Rumney (2007) examined the retention of ^210^Po from consuming shellfish and concluded its biological half-life was about 40 days, which is consistent with the International Commission on Radiological Protection (ICRP 1993) value of 50 days. On the other hand, Thomas et al. (2001) concluded that the biological half-life of ^210^Po in humans following consumption of caribou meat is > 100 days and that once absorbed ^210^Po is excreted very slowly if at all. Assuming that the biological half-life is 50 days, then the effective half-life in humans would be about 37 days.

*Biodistribution.* The tissue distribution of ^210^Po across mammalian species is broadly similar (Thomas et al. 2001). ^210^Po concentrations in the bones of farm animals are 2–3 orders of magnitude greater than in the muscles (Parfenov 1974). Average relative ^210^Po activities in the liver, kidney, and muscle were 19, 13, and 1, respectively, in caribou and 24, 19, and 1, respectively, in wolves (Thomas et al. 1994). In adult female baboons, the highest concentrations of injected ^210^Po were found in the liver and kidney, and the lowest were in the muscle, skeleton, and brain (Fellman et al. 1994).

Ingested ^210^Po in humans is initially concentrated in red blood cells, followed by the liver, kidneys, bone marrow, gastrointestinal tract, and gonads (Jefferson et al. 2009). Once absorbed into the blood, about 30% goes to the liver, 10% to red bone marrow, 10% to the kidney, 5% to the spleen, and the remainder to the body in general (Thomas et al. 2001). Average wet weight ^210^Po concentrations for nonsmokers were 0.055, 0.53, and 0.48 Bq/kg in skeletal muscle, liver, and kidney, respectively (Parfenov 1974). Based on reported measurements of ^210^Po in the various organs and the weights of the organs in the standard reference person, the total ^210^Po content of the human body was estimated to be about 20 Bq (Parfenov 1974).

Median wet weight ^210^Po concentrations from cattle in an uncontaminated agricultural area in Germany were 1.2 Bq/kg in the liver and 6.6 Bq/kg in the kidney (Bunzl et al. 1979). In northern Poland with background levels of radioactivity, Skwarzec and Prucnal (2007) found that the average wet weight ^210^Po concentrations in deer from these areas were 0.16 Bq/kg in muscle, 1.08 Bq/kg in liver, and 1.58 Bq/kg in kidney. Levels for animals from contaminated areas can be significantly higher. In the Arctic, lichens accumulate large amounts of ^210^Po from the atmosphere and are the main winter forage for caribou (Thomas et al. 2001). In Canadian caribou that consume the lichens, ^210^Po concentrations ranged from 9 to 40 Bq/kg in meat and from 90 to 620 Bq/kg in liver and kidney (Thomas et al. 2001). In cattle raised in an area in New Mexico that was frequently flooded by untreated effluent from a nearby uranium mine, Lapham et al. (1989) found that the average wet weight ^210^Po concentrations were 3.4, 56, and 65 Bq/kg in muscle, liver, and kidney, respectively.

In general, testes and ovaries contain elevated ^210^Po concentrations compared with most other tissues. An assessment of ^210^Po biokinetics suggested that transfer coefficients to the testes and ovaries are 0.1% and 0.05% per day, respectively, for ^210^Po in blood plasma that originated from the gastrointestinal tract or from systemic tissues returning to the blood (Leggett and Eckerman 2001). ^210^Po in the ovaries accumulates preferentially in the cells of the follicular epithelium and in the connective tissue cells of the corpora lutea (Moroz and Parfenov 1972). Ten days after injecting 925,000 Bq/kg into mice, Finkel et al. (1953) found that the ^210^Po concentrations in the ovaries were very high and caused severe damage to the ovaries. At 10 days, concentrations in the ovary were exceeded only by concentrations in the spleen; however, within 120 days after the injection, ^210^Po concentrations in the ovaries had decreased and had become undetectable.

Published values for ^210^Po concentration in cow’s milk ranged from 3.3 to 18.5 mBq/L and were > 1 order of magnitude lower than in meat (Parfenov 1974). Concentrations can be much higher in milk from areas known to be contaminated. For example, the geometric mean ^210^Po concentration in milk samples from cattle raised in a uranium-mining district in India was 1.08 Bq/L (Giri et al. 2010), with a maximum of 2.94 Bq/L.

After injecting ^210^Po into pregnant rats and guinea pigs, Haines et al. (1995) reported that fetal and placental tissues contained about 3–4% of the injected ^210^Po in rats and about 10% in guinea pigs. The authors also observed that at birth each rat pup had about 0.1% of the injected ^210^Po and that on day 57 each guinea pig fetus contained 0.6%. The highest concentrations of ^210^Po, about 4% of the injected ^210^Po per gram, were measured in the yolk sacs of the embryonic rats during their hematopoietic stage (Haines et al. 1995). The data suggest that the placenta acts as a barrier against movement of ^210^Po from mother to fetus. In one population of caribou in Canada, fetal ^210^Po averaged 62% of the ^210^Pb and 41% in a second population of caribou (Thomas et al. 1994). We calculated that within the caribou gestation period of 230 days, it would take 204 and 117 days, respectively, in these two populations, for the decay of ^210^Pb to generate this much ^210^Po. Thus, decay of fetal ^210^Pb could have provided all or most of the of the measured fetal ^210^Po in the caribou. Paquet et al. (1998) found that approximately 0.7–1% of ^210^Po injected into pregnant baboons at 5 months was present in the fetus 7 days later, which indicates that small amounts of ^210^Po do pass through the placenta. Söremark and Hunt (1966) found that the autoradiographs of mice 4 days after being injected with ^210^Po showed ^210^Po was present in the placenta but not in the fetus; however, by the fifth day, ^210^Po was detected in the fetus. By this time, the placenta was showing damage, which suggests that ^210^Po transport across the placenta may change with dose and time.

*Effects of ^210^Pb on ^210^Po biokinetics and biodistribution.* ^210^Pb biokinetics affect ^210^Po biokinetics and biodistribution because ^210^Pb decays to ^210^Po. Lead accumulates in skeletal bone: 67% of the lead burden in children is in bone and 95% in adults (Barry and Mossman 1970). ^210^Pb readily passes the placenta (Goyer 1990) and ^210^Pb-supported ^210^Po (^210^Po originating from *in situ* decay of ^210^Pb) can irradiate fetal tissues. ^210^Po produced by decay of skeletal ^210^Pb remains in the bone (James et al. 2004); however, ^210^Po from the decay of soft-tissue ^210^Pb likely would follow typical ^210^Po biokinetics. ^210^Po activity begins to increase after ^210^Pb ingestion and reaches 25% of the ^210^Pb activity in 65 days, 50% in 145 days, and approximately 99% of the ^210^Pb activity in 2 years. Because ^210^Po concentrations increase as ^210^Pb in the body decays, exposure of the fetus and children to ^210^Pb-supported ^210^Po would be least during early pregnancy and would increase with age.

## Toxicology of ^210^Po

Unlike some alpha emitters such as ^238^U, ^210^Po is toxic because of its radioactivity rather than its chemical properties (Jefferson et al. 2009). Acute lethal doses cause fatal damage to the bone marrow and, in addition, cause severe damage to the kidneys, spleen and gastrointestinal tract (Scott 2007). ^210^Po presents a radiation hazard only if taken into the body because its alpha particles have a range of only 40–50 µm in biological tissue and are easily stopped by surface layers of the skin (Jefferson et al. 2009).

^210^Po is a known human carcinogen (IARC 2001), and its carcinogenicity to animals has been demonstrated by numerous animal studies (reviewed by Moroz and Parfenov 1972). Exposure of the lungs to ^210^Po alone is capable of causing lung cancer in rats and hamsters (Little and O’Toole 1974; Yuile et al. 1967). Injecting ^210^Po into CF-1 female mice caused an increased incidence of lymphomas and soft-tissue and malignant-bone tumors (Finkel and Hirsch 1950). At a dose of 33.3 Bq/g body weight, 42% of autopsied mice had developed lymphomas within 250 days of injection compared with 21% of the controls.

Injected ^210^Po was taken up by the ovaries in mice and localized primarily in the follicle cells, whereas in the uterus it was distributed diffusely through the myometrium (Samuels 1966). Significant killing of primary oocytes occurred after exposure to as little as 0.037 Bq/g body weight ^210^Po, with higher doses killing almost all the oocytes and causing gross distortion of the ovarian architecture. Radioautographs led Samuels (1966) to conclude that ^210^Po appears to concentrate in the ovary in such a manner that it selectively irradiated the oocytes. Finkel et al. (1953) concluded that the ovary may be the critical organ in determining the lowest injurious dose for ^210^Po because of its radiosensitivity and tendency to concentrate ^210^Po.

For alpha radiation, substantial doses of radiation (0.5–1.3 Gy) are delivered to individual cells that are traversed by a single alpha particle no matter how low the dose to the whole body (Lorimore et al. 1998; Purnell et al. 1999). Alpha particles traversing the nucleus of HeLa cells produced a track averaging 10 µm, with an average of 22 double-strand chromosomal breaks (DSBs) (Aten et al. 2004). Breaks found at distant locations can subsequently be brought together and form chromosomal translocations (Aten et al. 2004). Alpha emitters can induce DNA lesions in stem cells that result in the transmission of chromosomal instability to their progeny (Kadhim et al. 1992), and even a single alpha particle can induce long-term chromosomal instability in primary human T lymphocytes (Kadhim et al. 2001). Chromosomal translocation and aneuploidy are a hallmark of the childhood leukemias regardless of specific histology (Szczepański et al. 2010). Because ^210^Po is an alpha emitter, toxicological investigations on the cellular and molecular effects of alpha radiation using other alpha emitters likely will also apply to ^210^Po. Irrespective of their source, alpha particles produce the same patterns of secondary ionizations and localized damage to biological molecules (IARC 2001), thus the important difference between different alpha emitters would be in what tissues they preferentially accumulate in, and thus what tissues are preferentially irradiated.

Radiation-induced bystander effects result when ionizing radiation, such as that from an alpha particle traversing a cell, causes targeted cells to produce signals that damage unirradiated neighboring cells (Hall 2003). Biological consequences to bystander cells include apoptosis, micronucleus induction, sister-chromatid exchange, chromosomal rearrangements, gene mutations, cell transformations, and delayed effects on their progeny such as genomic instability (Morgan and Sowa 2007). Bystander signals initiated by very low doses of alpha particles in irradiated normal human diploid fibroblasts induced DSB damage in unirradiated cells, and the percentage of DSBs in bystander cells was not dependent on the dose delivered (Hu et al. 2006). Exposure of only four human fibroblast cells to five alpha particles each was sufficient to induce a significant increase in micronucleated and apoptotic cells in a population of > 3,000 neighboring cells (Prise et al. 1998). In experiments where < 1% of the Chinese Hamster ovary cells in a culture were actually traversed by an alpha particle, Nagasawa and Little (1992) observed an increase in sister-chromatid exchange in > 30% of the cells. In a normal human three-dimensional skin-tissue system, Belyakov et al. (2005) saw significant increases in micronuclei formation and apoptosis in unirradiated cells up to 0.6 mm and 1.0 mm distant, respectively, from irradiated cells. The significance of the bystander effect for alpha particles is that linear extrapolation of the risk of carcinogenesis from high doses to low doses would underestimate the risks at low doses (Hall 2003).

## Direct and Indirect Ingestion of ^210^Po

In uncontaminated settings, only about 14% of the human body burden is due to direct ingestion of ^210^Po, principally in food, and the remainder is indirectly due to ingestion of ^210^Pb, which decays to ^210^Po (Parfenov 1974). In soft tissues, about half of the ^210^Po originates from ^210^Pb, whereas in bone almost all of the ^210^Po originates from ^210^Pb (Parfenov 1974). Atmospheric inhalation, diet, and domestic radon are estimated to provide 12%, 86%, and 2%, respectively, of total ^210^Pb uptake; however, in some locations, inhalation can be the largest source of ^210^Pb (Salmon et al. 1998). Smoking and consumption of alcoholic beverages can add an extra 75% to the total ^210^Pb uptake (Salmon et al. 1998). In the absence of ^210^Po ingestion or inhalation, the ^210^Po activity will be approximately 99% of the ^210^Pb activity within 2 years.

*Direct ingestion of ^210^Po-contaminated water.* It is unusual to find ^210^Po activities greater than approximately 30 mBq/L in groundwater because it is rapidly adsorbed onto particles (Harada et al. 1989); however, in some geochemical settings, much higher levels of ^210^Po are found (Seiler et al. 2011). Exposure to contaminated drinking water is particularly insidious because it may be chronic and unrecognized, especially in rural areas where the population relies on private wells that are unregulated and not tested for radioactivity. Analyses for the presence of ^210^Po in drinking water are rarely made, and ^210^Po is not measured directly to determine compliance with drinking-water standards for public supply wells in the United States. In the United States, compliance is assessed indirectly by analyzing the U activity and the gross alpha radioactivity (GAR), a measure of emitted alpha radiation that does not identify the specific isotopes involved. The water samples are evaporated to dryness, which drives off radon gas, and the standard is violated if the GAR of the sample exceeds the U activity by 0.55 Bq [U.S. Environmental Protection Agency (EPA) 2000].

^210^Po activities in private wells in Lahontan Valley, Nevada, were measured at levels as high as 6.59 Bq/L (Seiler 2011), and these private wells serve hundreds to thousands of people in areas where ^210^Po commonly exceeds 0.5 Bq/L. In one well, the ^210^Po activity was stable in measurements made six half-lives (830 days) apart (Seiler 2012), which suggests users of contaminated wells in Lahontan Valley have been exposed to ^210^Po since the wells were drilled. Several wells with high ^210^Po in West Central Florida were used as household drinking-water sources or for lowering groundwater levels near phosphate mines (Burnett et al. 1988). One intensively studied well in Hillsborough County, Florida, with ^210^Po activities ranging between 9.6 Bq/L and 95 Bq/L (Burnett et al. 1988) was a private well used by several families as their main source of drinking water until the ^210^Po contamination was discovered. Contaminated municipal wells have served large numbers of people for more than a decade before the contamination was discovered. For example, a municipal well in service since 1992 provided water for 95 houses in a subdivision in Charles County, Maryland, and was discovered to contain 1.7 Bq/L ^210^Po in 2004 (Outola et al. 2008). The presence of ^210^Po was not discovered earlier because before 2002 it took about 1 year before samples were analyzed (Outola et al. 2008), during which time ^210^Po was being lost through radioactive decay.

The residents of parts of Lahontan Valley, where ^210^Po in private wells exceeds 0.5 Bq/L, can potentially be exposed to substantial doses of radioactivity. Using the dose conversion factor of 1.2 × 10^–3^ mSv/Bq for ^210^Po 1 year after ingestion (ICRP 2010), the annual effective dose would be > 0.4 mSv/year for adults who consumed 2 L/day of water from wells with > 0.5 Bq/L of ^210^Po. For adults using the well with 6.59 Bq/L of ^210^Po, the annual effective dose would be almost 6 mSv/year. For comparison, the World Health Organization (WHO 2006) concluded that no deleterious radiological health effects are expected from consumption of drinking water if the committed effective dose (i.e., the total effective dose received over a lifetime), does not exceed 0.1 mSv/year.

Effective dose coefficients are higher for infants than for adults, in part because the fraction of ^210^Po absorbed from the alimentary tract into the blood, f1, is assumed to be 100% for a child < 1 year of age, and 50% for older ages (ICRP 2010). For a 3-month-old child, the effective dose coefficient is 2.6 × 10^–2^ mSv/Bq for ^210^Po 1 year after ingestion, and for a 1-year-old child, it is 8.8 × 10^–3^ mSv/Bq (ICRP 2010). For a 3-month-old child who consumed 1 L/day of well water used to prepare infant formula, the annual effective dose would be almost 5 mSv/year for well water that contained 0.5 Bq/L of ^210^Po and about 60 mSv/year for well water that contained 6.59 Bq/L of ^210^Po. For a 3-month-old child, the committed equivalent dose coefficient is 5.7 × 10^–3^ mSv/Bq for the ovary, 8.1 × 10^–2^ mSv/Bq for red bone marrow, and 2.2 × 10^–1^ mSv/Bq for the spleen (ICRP 2010). Therefore, a 3-month-old child who consumed 1 L/day of well water that contained 0.5 Bq/L of ^210^Po would receive doses to the ovary, red bone marrow, and spleen of about about 1, 15, and 40 mSv/year, respectively.

*Direct ingestion of ^210^Po-contaminated food.* A review of food-chain transport of ^210^Po concluded that the greatest sources of ingested ^210^Po were found in populations with large dietary complements of seafood (Japan) or caribou and reindeer muscle and organs (Arctic) (Watson 1985). In shellfish and crustaceans, Lee et al. (2009) reported ^210^Po concentrations ranging from 19.1 to 33 Bq/kg in Korea, and Carvalho (1995) reported levels from 49 to 152 Bq/kg in Portugal. Because all marine organisms accumulate ^210^Po in high concentrations due to its solubility in seawater and its affinity to organic matter, seafood is a notable source of ^210^Po in the human diet (Alam and Mohamed 2011).

Caribou, which are a main dietary staple for many northern Canadians, bioaccumulate ^210^Po from lichens—their main winter forage (Thomas and Gates 1999). The surfaces of lichens are highly efficient at capturing ^210^Pb and ^210^Po from atmospheric fallout, with concentrations of about 250 Bq/kg dry weight (Persson and Holm 2011). The average ^210^Po concentration in lichen samples from 20 sites in northern Saskatchewan, Canada, was 232 Bq/kg. Thomas and Gates (1999) collected samples from 18 caribou that had consumed the lichen and found that the average ^210^Po concentration in the liver, kidney, and muscles was 332 Bq/kg (wet weight), 156 Bq/kg, and 14 Bq/kg, respectively.

## Tobacco Toxicology and ^210^Po

Talhout et al. (2011) listed 98 hazardous compounds in mainstream cigarette smoke, including ^210^Po, and 51 other components that are known, probable, and possible human carcinogens. The ^210^Po concentration in tobacco has a mean ± SD of 13 ± 2 Bq/kg (Persson and Holm 2011), and the estimated daily inhalation of ^210^Po in smokers was between 13 and 590 mBq/day (Watson 1985). Zagà et al. (2011) concluded that ^210^Po is one of the most powerful carcinogens in tobacco smoke and, as previously noted, exposure of the lungs to ^210^Po alone is capable of causing lung cancer in rats and hamsters (Little and O’Toole 1974; Yuile et al. 1967).

Cigarette smoking has several effects that appear consistent with exposure to ^210^Po. A 60% increase in the risk of infertility among smokers may be related to ovarian toxicity (Augood et al. 1998). Cigarette smoking appears to significantly reduce ovarian reserves in women (El-Nemr et al. 1998), and Tuttle et al. (2009) found that the ovaries of mice that had been exposed to cigarette smoke were on average 20% smaller and had significantly fewer follicles. These outcomes appear similar to those described by Samuels (1966) for mice exposed to ^210^Po. Cigarette smoking also significantly increases a woman’s risk of developing mucinous ovarian cancer (Gram et al. 2011; Jordan et al. 2006). This may be linked to exposure to ^210^Po, which is known to accumulate in the ovary (Finkel et al. 1953).

Cigarette smoking has been linked to hematopoietic cancers, and a possible, although unstudied, contributing factor could be ^210^Po. For example, a case–control study on childhood cancer (Stjernfeldt et al. 1986) concluded that the risk of non-Hodgkin lymphoma and acute lymphoblastic leukemia (ALL) were doubled in the offspring of women who smoked > 10 cigarettes per day. Benzene is clearly a leukemogen in adults (Schnatter et al. 2005) and is typically indicated as the primary leukemogen in cigarettes. The contribution of tobacco carcinogens to leukemia, however, is predicated by an ability to target the appropriate organs, and ^210^Po shares with benzene a tropism to the bone marrow as well as fetal hematopoietic organs. To be sure, ^210^Po is not likely to be the major constituent in cigarette smoke’s contributions to leukemogenesis, but constitutes a dose- and mechanism-relevant constituent that deserves further scrutiny.

## ^210^Po and Leukemia

*Leukemogenesis.* The best substantiated cause for acute leukemia is via ionizing radiation (Greaves 1997), and recently published risk models suggest that about 15% of childhood leukemia in Great Britain is associated with natural background radiation (Kendall et al. 2011). Although experimental data are limited, the disposition and biological activities of ^210^Po in human and animal tissues are consistent with a role as a myelotoxicant and leukemogen. Those adult leukemias epidemiologically associated with chronic exposure to cigarettes or contaminated drinking water have cytogenetic characteristics that are typical of an alpha emitter such as ^210^Po. These leukemias commonly harbor broken chromosomes as well as aneuploidy, including loss of the q arm of chromosomes 5 and 7 and complete loss of chromosome 7 (Pedersen-Bjergaard and Rowley 1994).

^210^Po is equally plausible as a leukemogen in children. Epidemiologic studies on atomic bomb survivors indicated that young children were much more sensitive to radiation-induced leukemia than were adults (Little 2003). ^210^Po accumulates in several tissues with the capacity for hematopoiesis, including the fetal yolk sac and bone marrow and liver in mature animals as explained above. Both ALL and acute myelocytic leukemia (AML) in children are known to originate in the fetus, as demonstrated by the presence of cells with leukemia clone-specific mutations present at birth in children who later contract the disease (Wiemels 2008). Indirect evidence for the fetal origin of childhood ALL stems from the immature nature of immunoglobulin rearrangements as well as lack of evidence for recombinase activating gene-induced alterations at chromosomal translocations (Steenbergen et al. 1994; Wasserman et al. 1992). These translocations as well as chromosomal deletions and aneuploidy are the characteristic hallmark of childhood ALL (Greaves and Wiemels 2003; Mullighan et al. 2007). Translocations are the primary method of dosimetry of ionizing radiation exposure (Tucker 2008). Ionizing radiation from sources such as alpha particles has the capacity to both break DNA, a precursor to translocations and deletions (Cohen-Jonathan et al. 1999), and to elicit mitotic catastrophe, a type of cell death that occurs during mitosis (Castedo et al. 2004). Cells that fail to execute apoptosis in response to mitotic failure are likely to divide asymmetrically, with the consequent generation of aneuploid cells (Castedo et al. 2004). It is notable that up to 35% of childhood leukemias exhibit “high hyperdiploidy,” which is defined as having five or more extra chromosomes; this cytogenetic feature is known to occur after a single mitotic failure *in utero* (Gruhn et al. 2008; Paulsson et al. 2005).

*Lahontan Valley childhood leukemia cluster.* Between 1997 and 2002, 15 cases of ALL and 1 case of AML were diagnosed in children who lived in Lahontan Valley and in the city of Fallon in Churchill County, Nevada, before their diagnosis (Rubin et al. 2007). In the year 2000 alone, 9 cases of ALL were diagnosed in this county of only 26,000 people. Because > 12 cases were diagnosed during a 4-year period in which fewer than 2 cases would be expected, the U.S. Centers for Disease Control and Prevention led a multiagency effort to conduct a comprehensive cross-sectional exposure assessment that began in March 2001 (Rubin et al. 2007). Most research on the leukemia cluster focused on chemical exposures such as pesticides, jet fuel, volatile organic compounds, radon, arsenic, and tungsten (Rubin et al. 2007; Seiler 2004); however, no plausible exposure was identified. Instead, the temporal and spatial patterning of the leukemia cluster suggested involvement of an infectious disease (Francis et al. 2012). The temporal and spatial patterning was inconsistent with exposure to ^210^Po in drinking water (Seiler 2012); however, other possible sources of ^210^Po exposure were not investigated.

## Discussion and Research Needs

Animal research studies indicate that ^210^Po accumulates in the reproductive and blood-forming organs and that some ^210^Po is probably transferred from the mother to the embryo and fetus where its high-energy alpha particles can potentially kill or damage critical cells. These effects are consistent with exposure to ^210^Po in cigarettes because maternal smoking is known to affect maternal fertility and possibly the development of cancer in offspring. Although ^210^Po is not the only carcinogen in cigarettes, daily inhalation of ^210^Po by smokers exposes them to between 0.013 and 0.59 Bq/day (Watson 1985). Relatively small amounts of ^210^Po in drinking water near the current U.S. standard (U.S. EPA 2000), or in food, could result in greater exposure to women to ^210^Po than from smoking, and subtle unrecognized health effects may be occurring in women who are exposed to environmental ^210^Po.

Little is specifically known about the effects of ^210^Po exposure to the embryo and fetus, however, fetal exposure could cause failure of implantation or miscarriage or major malfunctions (Jefferson et al. 2009), as well an increased incidence of chromosomal breaks and translocations resulting in childhood cancer. At particular stages of development a future tissue, or stem cells for many cell lineages, can be represented by one or a few cells, and alpha irradiation may cause mutagenic effects in these cells that is later manifested as cancer (Goodhead 1991). The distribution of ^210^Po to the ovaries and the embryo (Finkel et al. 1953; Haines et al. 1995; Samuels 1966) and the potential for a single alpha particle traversing a stem cell to induce chromosomal instability (Kadhim et al. 2001) suggests that even low-level alpha-particle irradiation to reproductive or embryonic cells may have significant downstream effects. New, basic, biological, and toxicological research is needed on ^210^Po because of its potential to kill or damage critical reproductive, embryonic, or hematopoietic cells at low doses. New research is needed on transplacental transfer of ^210^Po and the effects of low-level exposure to the embryo, fetus, and infant because the high degree of cell kinetics needed for normal growth of embryonic and hematopoietic cells and other tissues in the fetus and child imparts a high sensitivity to children from environmental insults such as ionizing radiation from ^210^Po.

^210^Po may be involved in leukemogenesis by exposing the hematopoietic stem cells in the embryonic yolk sac and fetal spleen, liver, and bone marrow to alpha-particle radiation. The placenta provides at least a partial barrier to transfer of ^210^Po; however, ^210^Pb readily passes the placenta (Goyer 1990). Fetal ^210^Pb is incorporated into bone and ^210^Pb-supported ^210^Po could irradiate hematopoietic stem cells in fetal marrow. ^210^Po remains in place following decay of calcified ^210^Pb (James et al. 2004), and up to 80% of the marrow in lumbar vertebrae would be within range of alpha particles from ^210^Po on bone surfaces because bone-marrow spaces are small in the fetus (Purnell et al. 1999). Maternal ^210^Po that passes the placenta, or ^210^Pb-supported ^210^Po in soft tissues, could migrate in the blood to the fetal liver or spleen and potentially expose hematopoietic stem cells. Fat is present in adult but not fetal bone marrow and, being fat soluble, inhaled or ingested radon could accumulate in fat in adult marrow and expose it to alpha-particle irradiation from the radon itself or its daughters ^218^Po and ^214^Po (Richardson et al. 1991). New research is needed on toxicological and health effects of adult and embryonic and fetal exposure to Po isotopes, regardless of whether the exposure originates from ingestion of ^210^Po itself or its radionuclide grandparents radon and ^210^Pb. The emergence of exquisitely sensitive modern leukemia mouse models should be used to explore mechanisms of carcinogenesis by ^210^Po and ^210^Pb administered by appropriate routes and doses. Such models are currently used primarily to explore genetic pathways but increasingly are being made available for environmental research.

The linkage between smoking and female infertility (Augood et al. 1998) and the smaller size and reduced number of follicles in the ovaries of mice exposed to cigarette smoke (Tuttle et al. 2009) appear similar to outcomes described by Samuels (1966) for mice where primary oocytes were killed at low ^210^Po doses and the structure of the ovaries affected at higher doses. An unrecognized outcome for women exposed to environmental ^210^Po may be subfertility. Dose calculations together with a cell-survival function for primary oocytes could be used to estimate doses to the ovary and provide information on the expected level of killing of reproductive cells by environmental levels of ^210^Po. A single alpha particle can induce bystander effects in a population of cells, thus the sensitivity of primary oocytes to low doses of ^210^Po noted by Samuels (1966) may be the result of bystander effects. There may be tissue-specific differences in bystander responses, and research is needed on the susceptibility of reproductive and embryonic cells to bystander signals.

Numerous epidemiological studies reviewed by Canu et al. (2011) have investigated associations between cancers, including leukemia, and ingestion of naturally radioactive water. Even if exposure to ^210^Po was not occurring, ^210^Po must have been present in the environment at these studies because ^238^U, ^226^Ra, and radon ultimately decay to ^210^Po. Future investigations of natural radioactivity should consider the possibility that exposure to ^210^Po is occurring.

Residents of the subdivision in Charles County, Maryland (Outola et al. 2008), and rural areas of Lahontan Valley, Nevada (Seiler 2012), were exposed to levels of ^210^Po exceeding 1.5 Bq/L in their drinking water for decades before it was discovered. If the ^210^Po activity exceeds 0.55 Bq/L, then the GAR standard for ^210^Po would be exceeded (U.S. EPA 2000). Hence, even levels that meet the current drinking-water standard could expose the public to more ^210^Po than they would receive if they were smokers. The occurrence of ^210^Po concentrations in groundwater > 0.55 Bq/L is currently assumed to be extremely rare, but this rarity may be an artifact of groundwater seldom being directly tested for ^210^Po. Furthermore, the standard test for GAR (U.S. EPA 2000) may not adequately identify samples with ^210^Po concentrations that exceed safety thresholds. The recommended procedure allows quarterly samples to be composited as long as they are analyzed within 1 year of the first sample being collected (U.S. EPA 2000). However, during this 1-year period, > 60% of the ^210^Po in the composited samples can be lost by radioactive decay before analysis. Furthermore, sample heating during the analytical procedure for gross alpha radioactivity can drive off volatile radionuclides such as ^210^Po (Arndt and West 2008). Research on the environmental occurrence of ^210^Po and ^210^Pb is needed, particularly in drinking water, because of potential health effects at low levels. Development of new, less expensive, analyses for ^210^Po and ^210^Pb is needed because of the potential that current screening tests for them do not adequately protect public health.

Additional research on the occurrence of ^210^Po in groundwater and the f1 of naturally occurring ^210^Po is also needed because of the potential for contaminated food chains resulting from contaminated wells being used as water supplies for food animals. Chemical or radiological testing of water used for stock animals is not required by state or federal governments, and testing of meat and dairy products for contaminants found in animal’s stockwater is not required by the U.S. Food and Drug and Administration (FDA). When the discovery of ^210^Po in Lahontan Valley was first reported, two dairies that used wells with > 2 Bq/L ^210^Po were required to dump thousands of gallons of milk by the local dairy cooperative until the FDA could test whether the milk was safe. Samples of raw milk, beef, liver, and kidney from the dairies were analyzed by the FDA (Lin and Wu 2009) and the FDA concluded that the milk was safe (Associated Press 2007). However, the analytical results and risk analyses were never released to the public.

## Summary

The extent of environmental exposure to ^210^Po is poorly known, and environmentally relevant exposure to it may have subtle unrecognized health effects that merit further investigation because ^210^Po accumulates in reproductive and embryonic and fetal tissues where a single alpha particle can kill or damage critical cells. ^210^Po is present in cigarettes, and maternal smoking is known to affect ovarian reserve and possibly the development of cancer in the offspring. Daily exposure from ^210^Po in cigarettes is less than a person could receive from drinking water that meets U.S. drinking-water standards (U.S. EPA 2000), which raises the possibility that subtle health effects similar to those observed in women who smoke might be observed in exposed members of the public.

In uncontaminated settings, only about 14% of the human body burden is due to direct ingestion of ^210^Po; the remainder is indirectly due to ingestion of ^210^Pb. Nonetheless, exposure to ^210^Po through drinking water may be more widespread than currently assumed and a comprehensive national reconnaissance is needed to determine what levels of ^210^Po and ^210^Pb the U.S. population is being exposed to in its drinking water. ^210^Po concentrations in typical aquifer sediments are high enough that mobilization of only a small percentage of the ^210^Po present in the sediments could yield water with sufficient ^210^Po to affect the health of people drinking the water. For adults and infants using wells with 0.5 Bq/L of ^210^Po, which would meet the current U.S. drinking-water standard, the annual effective dose would exceed the limit of 0.1 mSv/year recommended by the WHO (2006). Private wells used in rural areas and wells used for stockwater are unregulated, and even gross ^210^Po contamination of one of these wells would almost never be detected.

Much of the important biological and toxicological research on ^210^Po is more than four decades old. Modern tools are required to assess environmentally relevant exposure to ^210^Po and its capacity to kill or damage critical reproductive, embryonic, or hematopoietic cells in humans. We call attention to these research needs to assess whether current regulatory standards to protect public health are appropriate so effective mitigation measures can be developed if necessary.
